# Modeling the architecture of the regulatory system controlling methylenomycin production in *Streptomyces coelicolor*

**DOI:** 10.1186/s13036-017-0071-6

**Published:** 2017-10-03

**Authors:** Jack E. Bowyer, Emmanuel LC. de los Santos, Kathryn M. Styles, Alex Fullwood, Christophe Corre, Declan G. Bates

**Affiliations:** 1 Warwick Integrative Synthetic Biology Centre, School of Engineering, University of Warwick, Coventry, CV4 7AL UK; 2 Warwick Integrative Synthetic Biology Centre, Department of Chemistry, University of Warwick, Coventry, CV4 7AL UK; 3 School of Life Sciences, University of Warwick, Coventry, CV4 7AL UK; 4 Warwick Integrative Synthetic Biology Centre, Department of Chemistry and School of Life Sciences, University of Warwick, Coventry, CV4 7AL UK

**Keywords:** Synthetic biology, Antibiotics, Gene regulation, Methylenomycin, Mathematical modelling, Approximate Bayesian computation, Global optimization

## Abstract

**Background:**

The antibiotic methylenomycin A is produced naturally by Streptomyces coelicolor A3(2), a model organism for streptomycetes. This compound is of particular interest to synthetic biologists because all of the associated biosynthetic, regulatory and resistance genes are located on a single cluster on the SCP1 plasmid, making the entire module easily transferable between different bacterial strains. Understanding further the regulation and biosynthesis of the methylenomycin producing gene cluster could assist in the identification of motifs that can be exploited in synthetic regulatory systems for the rational engineering of novel natural products and antibiotics.

**Results:**

We identify and validate a plausible architecture for the regulatory system controlling methylenomycin production in S. coelicolor using mathematical modeling approaches. Model selection via an approximate Bayesian computation (ABC) approach identifies three candidate model architectures that are most likely to produce the available experimental data, from a set of 48 possible candidates. Subsequent global optimization of the parameters of these model architectures identifies a single model that most accurately reproduces the dynamical response of the system, as captured by time series data on methylenomycin production. Further analyses of variants of this model architecture that capture the effects of gene knockouts also reproduce qualitative experimental results observed in mutant S. coelicolor strains.

**Conclusions:**

The mechanistic mathematical model developed in this study recapitulates current biological knowledge of the regulation and biosynthesis of the methylenomycin producing gene cluster, and can be used in future studies to make testable predictions and formulate experiments to further improve our understanding of this complex regulatory system.

## Background

There is currently an increasing demand for research and development of new antibiotics as their overuse, along with many other factors, has led to increased resistance. Streptomycetes produce approximately 70% of all commercial antibiotics currently available [1]. The bacterium *Streptomyces coelicolor* A3(2) has emerged as the model organism for studying streptomycetes, initially thanks to the production of colored metabolites that facilitated genetic studies, and more recently thanks to the sequencing of its entire genome [2]. These bacteria have a 8,667,507 base pair single linear chromosome containing protein coding genes of which over 12% are thought to be regulatory [2]. These predicted transcriptional regulators are thought to mediate antibiotic synthesis through the production of microbial hormones, as well as influence structural and metabolic cellular responses [3]. The linear SCP1 plasmid (∼356 kb) and the circular SCP2 plasmid (∼31 kb) are both present within the *S. coelicolor* genome and have also both been sequenced [4]. This genome sequencing has revealed many cryptic and ‘silent’ gene clusters: sets of genes predicted to produce a natural product, but whose product has not been observed. Silent gene clusters have been awakened through genetic manipulation of regulatory elements [5, 6]. Thus, characterization of the regulatory system that mediates the production of specialized metabolites is key to discovering new natural products. Developing improved understanding of the regulatory architectures that underlie natural product biosynthesis can also accelerate the design of novel regulatory systems in synthetic biology.

**Fig. 1:**
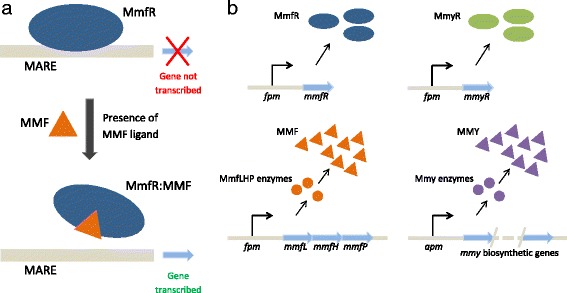
**a** Schematic diagram of the MmfR binding mechanism. Binding of MmfR to DNA at the MARE represses gene transcription and therefore negates system output. In the presence of MMF ligand, an MmfR:MMF complex is formed which releases MmfR from the MARE and triggers gene transcription. **b** Schematic diagram of the methylenomycin gene cluster whereby *fpm* and *apm* represent the DNA binding motifs recognized by MmfR and MmyR proteins. The *fpm* controls the expression of *mmfR*, *mmyR* and *mmfLHP* genes while *apm* regulates the expression of the *mmy* biosynthetic genes

The antibiotic methylenomycin A is a natural product of *S. coelicolor* A3(2) and is of particular interest since all of the 21 biosynthetic, regulatory and resistance genes, located in a cluster on the SCP1 plasmid [4, 7], have been studied in detail [8], and a series of knockout mutant strains has been generated [9]. The regulation of methylenomycin biosynthesis is mediated by the transcriptional repressor MmfR, a TetR-family homodimeric protein consisting of an N-terminal DNA-binding domain and a C-terminal ligand-binding domain (Fig. 1a) [10, 11]. In the initial growth phase of *S. coelicolor*, the MmfR N-terminal domain is thought to be bound to the DNA at the methylenomycin auto-regulatory response element (MARE) causing the transcriptional repression of downstream genes. MmfR holds the system in this repressed state until the advent of the small signaling molecules, methylenomycin furans (MMFs) [12]. MMFs bind specifically to the C-terminal domain of the MmfR, forming an MmfR:MMF complex that results in a conformational change in the MmfR. Consequently, MmfR is released from the MARE, negating the repression and triggering gene transcription. The biosynthesis of MMFs is controlled by the MmfLHP enzymes which are, themselves, repressed by MmfR, thus forming a feedback control loop that governs the dynamical properties of the system. A second repressor, MmyR, is homologous to MmfR yet its role in methylenomycin regulation is currently less understood. There is, however, clear evidence that the impact of MmyR is particularly significant, since *S. coelicolor* strains with the *mmyR* gene knocked out have been found to overproduce methylenomycin [9].

Homologous architectures to that of the methylenomycin regulatory system have been identified across a plethora of microorganisms [13], regulating different classes of natural products and thus indicating the utility of this specific type of regulatory architecture [12]. Responding to environmental changes is of paramount importance to these bacteria. The soil they live in presents a harsh environment with considerable competition for resources and it is therefore vital that they possess sophisticated, tightly regulated mechanisms to turn on the expression of specific genes when required. Hence, obtaining a detailed mechanistic understanding of the regulatory system controlling the biosynthetic pathway to this antibiotic has the potential to elucidate a host of other, less tractable, biosynthetic gene clusters and help standardize one of the most important regulatory networks for the development of new antibiotics.

Recent mathematical modeling investigations have generated new insights into the operation of numerous systems of interest to synthetic biologists [14–17]. Such models not only provide the capability to accurately simulate synthetic systems during the design and development phase, but are also effective tools for the prediction of system responses to variations in environmental conditions [18]. Here, we develop the first detailed mathematical model of the MMF-dependent regulatory system involved in methylenomycin production in *S. coelicolor*, firstly, through a rigorous statistical analysis of the plausibility of multiple candidate model architectures and, secondly, via global optimization of the relevant model parameters against available experimental data. We also validate our candidate model architecture against a range of selection criteria devised in light of experimental observations on methylenomycin production in several mutant *S. coelicolor* strains.

## Results and discussion

### Formulation of candidate model architectures

The various binding interactions and protein expression summarized in Fig. 1 inform the formulation of our candidate model architectures. MmfR is thought to bind to three distinct intergenic regions on the gene cluster [9]. However, we combine the region associated with MmyR biosynthesis together with the region associated with both MmfR and MMF biosynthesis to form a single DNA module responsible for the biosynthesis of all three molecules (the furan producing module, *fpm*). That is, we use the term *fpm* to refer to five distinct genes that provide control over three distinct molecular products: MmyR, MmfR and MMF. The genes *mmfL*, *mmfH* and *mmfP* are coregulated in an operon and are directly responsible for the production (assembly) of MMF molecules; the *mmfR* and *mmyR* genes control MmfR and MmyR production respectively [9, 12] (Fig. 1b). The third distinct intergenic region is represented by our second DNA module which we consider responsible for methylenomycin (MMY) biosynthesis only (the antibiotic producing module, *apm*). Therefore, our model architectures all consist of two fundamental DNA modules that can both be bound by MmfR, and that have production of their respective proteins repressed as a consequence. Due to its effect on the gene cluster and its homology to MmfR, in this study we hypothesize that MmyR also binds both modules in a similar manner.

**Fig. 2:**
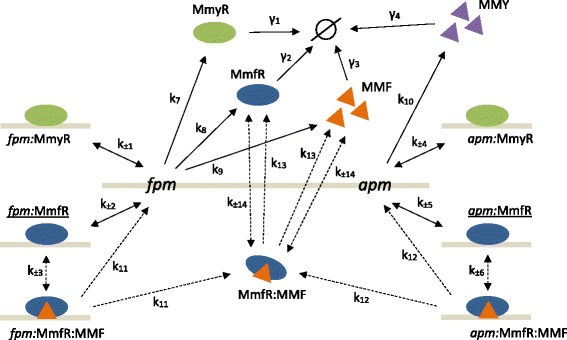
Schematic diagram of the reaction network comprising the base (BNN) model architecture. Reversible and irreversible reactions are depicted by double and single *arrows* respectively; reaction rate constants are denoted by the corresponding numbered k. The empty set depicts protein degradation, with rate constants denoted by the corresponding numbered *γ*. *Solid arrows* depict reactions that are common to all 48 model architectures, whereas *dashed arrows* depict those that are subject to adaptation. Cellular entities with non-zero initial concentrations are underlined

Our base architecture accounts for reversible MmfR and MmyR binding to both the *fpm* and *apm* to form four complexes: *fpm*:MmfR, *fpm*:MmyR, *apm*:MmfR and *apm*:MmyR. MMF binds MmfR reversibly at these complexes in order to trigger gene expression; MMF binding MmfR in solution is also accounted for since we have been able to co-crystallize MmfR:MMF complexes and solve the 3D-structure through experimentation void of target DNA modules (data not shown). MmfR:MMF complexes that dissociate from the MAREs return free MmfR and MMF back into the system irreversibly. MmyR, MmfR and MMF production is controlled by the *fpm*. We account for an initial repressed system state by imposing non-zero initial concentrations upon the *fpm*:MmfR and *apm*:MmfR complexes; all remaining model variables have initial concentrations equal to zero. MmfR, MmyR, MMF and MMY all undergo degradation at constant rates (Fig. 2).

This model architecture represents the extent of our current mechanistic understanding, however there are certain details that require further investigation. For example, although we believe that the MMF releases MmfR from the *fpm*:MmfR and *apm*:MmfR complexes and also binds free MmfR in solution, it would be insightful to examine the dynamical influence of each binding mechanism in isolation. Similarly, although we believe there is no interaction between the MMF and MmyR within the system (data not shown), the binding interactions of MMY are not as well documented. It may therefore be possible that MMY is able to inhibit the action of both MmfR and MmyR either through dissociation from their respective *fpm* and *apm* complexes or binding in solution. Consequently, the aim of our modeling investigation is to examine the effect of three key mechanistic properties on model performance:

1. MMF-MmfR interactions occur at existing DNA:MmfR complexes (C), in solution (S) or via both mechanisms (B).
2. MMY-MmfR interactions occur at existing DNA:MmfR complexes (C), in solution (S), via both mechanisms (B) or do not occur at all (N).
3. MMY-MmyR interactions occur at existing DNA:MmyR complexes (C), in solution (S), via both mechanisms (B) or do not occur at all (N).

This set of possible molecular interactions results in 48 distinct candidate model architectures for the methylenomycin regulatory system. Each candidate architecture is given a three letter name corresponding to the interactions accounted for with respect to each of the three properties listed above. The order of the letters in each name corresponds strictly to the numerical order of these properties. For example, our base architecture (described above) is given the name BNN since it accounts for both mechanisms (B) regarding property 1, for no interactions at all (N) regarding property 2 and for no interactions at all (N) regarding property 3.

Each of the 48 candidate architectures presents a distinct reaction network and set of biochemical equations that can be used to derive a dynamical mathematical model. We apply mass action kinetics to the biochemical equations comprising each reaction network to derive a corresponding system of ordinary differential equations (ODEs). Each ODE describes the rate of change in concentration corresponding to each model variable (cellular entity). The solution to each system of ODEs is determined numerically due to the non-linearity of the equations and provides a deterministic output that can be used to simulate and predict in vivo system dynamics in silico. For example, the BNN model architecture is comprised of the following biochemical equations:

1 $$\begin{array}{*{20}l} fpm + \text{MmyR} &\rightleftharpoons[k_{-1}]{k_{1}} fpm\text{:MmyR} \end{array} $$

2 $$\begin{array}{*{20}l} fpm + \text{MmfR} &\rightleftharpoons[k_{-2}]{k_{2}} fpm\text{:MmfR} \end{array} $$

3 $$\begin{array}{@{}rcl@{}} \textit{fpm} &\xrightarrow{k_{7}}& \text{MmyR} + \textit{fpm} \end{array} $$

4 $$\begin{array}{@{}rcl@{}} \textit{fpm} &\xrightarrow{k_{8}}& \text{MmfR} + \textit{fpm} \end{array} $$

5 $$\begin{array}{@{}rcl@{}} \textit{fpm} &\xrightarrow{k_{9}} &\text{MMF} + \textit{fpm} \end{array} $$

6 $$\begin{array}{*{20}l} fpm\text{:MmfR} + \text{MMF} &\rightleftharpoons[k_{-3}]{k_{3}} fpm\text{:MmfR:MMF} \end{array} $$

7 $$\begin{array}{@{}rcl@{}} \textit{fpm}\text{:MmfR:MMF} &\xrightarrow{k_{11}}& \textit{fpm} + \text{MmfR:MMF} \end{array} $$

8 $$\begin{array}{*{20}l} apm + \text{MmyR} &\rightleftharpoons[k_{-4}]{k_{4}} apm\text{:MmyR} \end{array} $$

9 $$\begin{array}{*{20}l} apm + \text{MmfR} &\rightleftharpoons[k_{-5}]{k_{5}} apm\text{:MmfR} \end{array} $$

10 $$\begin{array}{@{}rcl@{}} \textit{apm} &\xrightarrow{k_{10}}& \text{MMY} + \textit{apm} \end{array} $$

11 $$\begin{array}{*{20}l} apm\text{:MmfR} + \text{MMF} &\rightleftharpoons[k_{-6}]{k_{6}} apm\text{:MmfR:MMF} \end{array} $$

12 $$\begin{array}{@{}rcl@{}} \textit{apm}\text{:MmfR:MMF} &\xrightarrow{k_{12}}& \textit{apm} + \text{MmfR:MMF} \end{array} $$

13 $$\begin{array}{@{}rcl@{}} \text{MmfR:MMF} &\xrightarrow{k_{13}}& \text{MmfR} + \text{MMF} \end{array} $$

14 $$\begin{array}{*{20}l} \text{MmfR} + \text{MMF} &\rightleftharpoons[k_{-14}]{k_{14}} \text{MmfR:MMF} \end{array} $$

15 $$\begin{array}{@{}rcl@{}} \text{MmyR} &\xrightarrow{\gamma_{1}}& \varnothing \end{array} $$

16 $$\begin{array}{@{}rcl@{}} \text{MmfR} &\xrightarrow{\gamma_{2}}& \varnothing \end{array} $$

17 $$\begin{array}{@{}rcl@{}} \text{MMF} &\xrightarrow{\gamma_{3}} &\varnothing \end{array} $$

18 $$\begin{array}{@{}rcl@{}} \text{MMY} &\xrightarrow{\gamma_{4}}& \varnothing \end{array} $$

from which we derive the following system of model ODEs:

19 $$  {{\begin{aligned} \frac{d[\text{MmyR}]}{dt} &= k_{7}[fpm] - k_{1}[\text{MmyR}][fpm]+ \,k_{-1}[fpm\text{:MmyR}] \\ &\quad- k_{4}[\text{MmyR}][apm] + k_{-4}[apm\text{:MmyR}]\\ &\quad - \gamma_{1}[\text{MmyR}], \end{aligned}}}  $$

20 $$  {{\begin{aligned} \frac{d[\text{MmfR}]}{dt} &= k_{8}[fpm] - k_{2}[\text{MmfR}][fpm]+\, k_{-2}[fpm\text{:MmfR}]\\ &\quad - k_{5}[\text{MmfR}][apm] + k_{-5}[apm\text{:MmfR}]\\ &\quad + k_{11}[fpm\text{:MmfR:MMF}] + k_{12}[apm\text{:MmfR:MMF}] + \\ &\quad k_{13}[\text{MmfR:MMF}] - k_{14}[\text{MmfR}][\text{MMF}]\\ &\quad + k_{-14}[\text{MmfR:MMF}] - \gamma_{2}[\text{MmfR}], \end{aligned}}}  $$

21 $$  {{\begin{aligned} \frac{d[fpm]}{dt} &= k_{11}[fpm\text{:MmfR:MMF}] - k_{1}[\text{MmyR}][fpm]\\ &\quad + k_{-1}[fpm\text{:MmyR}] + k_{-2}[fpm\text{:MmfR}]\\ &\quad - k_{2}[\text{MmfR}][fpm], \end{aligned}}}  $$

22 $$  {{\begin{aligned} \frac{d[apm]}{dt} &= k_{12}[apm\text{:MmfR:MMF}] - k_{4}[\text{MmyR}][apm]\\ &\quad + k_{-4}[apm\text{:MmyR}] + k_{-5}[apm\text{:MmfR}]\\ &\quad - k_{5}[\text{MmfR}][apm], \end{aligned}}}  $$

23 $$  {{\begin{aligned} \frac{d[fpm\text{:MmyR}]}{dt} &= k_{1}[\text{MmyR}][fpm] - k_{-1}[fpm\text{:MmyR}], \end{aligned}}}  $$

24 $$  {{\begin{aligned} \frac{d[apm\text{:MmyR}]}{dt} &= k_{4}[\text{MmyR}][apm] - k_{-4}[apm\text{:MmyR}], \end{aligned}}}  $$

25 $$  {{\begin{aligned} \frac{d[fpm\text{:MmfR}]}{dt} &= k_{2}[\text{MmfR}][fpm] - k_{-2}[fpm\text{:MmfR}]\\ &\quad - k_{3}[fpm\text{:MmfR}][\text{MMF}] + \\ &\quad k_{-3}[fpm\text{:MmfR:MMF}], \end{aligned}}}  $$

26 $$  {{\begin{aligned} \frac{d[apm\text{:MmfR}]}{dt} &= k_{5}[\text{MmfR}][apm] - k_{-5}[apm\text{:MmfR}]\\ &\quad - k_{6}[apm\text{:MmfR}][\text{MMF}] + \\ &\quad k_{-6}[apm\text{:MmfR:MMF}], \end{aligned}}}  $$

27 $$  {{\begin{aligned} \frac{d[fpm\text{:MmfR:MMF}]}{dt} &= k_{3}[fpm\text{:MmfR}][\text{MMF}]\\ &\quad - k_{-3}[fpm\text{:MmfR:MMF}]\\ &\quad - k_{11}[fpm\text{:MmfR:MMF}], \end{aligned}}}  $$

28 $$  {{\begin{aligned} \frac{d[apm\text{:MmfR:MMF}]}{dt} &= k_{6}[apm\text{:MmfR}][\text{MMF}]\\ &\quad - k_{-6}[apm\text{:MmfR:MMF}]\\ &\quad - k_{12}[apm\text{:MmfR:MMF}], \end{aligned}}}  $$

29 $$  {{\begin{aligned} \frac{d[\text{MMF}]}{dt} &= k_{9}[fpm] - k_{3}[fpm\text{:MmfR}][\text{MMF}]\\ &\quad + k_{-3}[fpm\text{:MmfR:MMF}] + k_{-6}[apm\text{:MmfR:MMF}]\!\\ &\quad - k_{6}[\!apm\text{:MmfR}]\![\!\text{MMF}]+ k_{11}[fpm\text{:MmfR:MMF}] + \\ &\quad k_{12}[apm\text{:MmfR:MMF}] + k_{13}[\text{MmfR:MMF}]\\ &\quad - k_{14}[\text{MmfR}][\text{MMF}] + k_{-14}[\text{MmfR:MMF}]\\ &\quad - \gamma_{3}[\text{MMF}], \end{aligned}}}  $$

30 $$ {{\begin{aligned} \frac{d[\text{MMY}]}{dt} = k_{10}[apm] - \gamma_{4}[\text{MMY}], \end{aligned}}}   $$

31 $$  {{\begin{aligned} \frac{d[\text{MmfR:MMF}]}{dt} &= k_{11}[fpm\text{:MmfR:MMF}] + k_{12}[apm\text{:MmfR:MMF}]\\ &\quad - k_{13}[\text{MmfR:MMF}] + k_{14}[\text{\!MmfR}][\!\text{MMF}]\\ &\quad - k_{-14}[\!\text{MmfR:MMF}], \end{aligned}}}  $$

where square brackets denote concentration and the reaction rate constants translate to model parameters. Reactions associated with reversible DNA:protein binding (*k*_1_, *k*_−1_, *k*_2_, *k*_−2_, *k*_4_, *k*_−4_, *k*_5_ and *k*_−5_), the production of MmyR, MmfR, MMF and MMY (*k*_7_, *k*_8_, *k*_9_ and *k*_10_) and each individual protein degradation reaction (*γ*_1,2,3,4_) are common to all of our candidate model architectures. Other reactions that are associated with the release of MmfR from existing DNA:MmfR complexes or the sequestration of MmfR and MmyR via binding in solution are not common to all models and are thus subject to investigation through our computational simulations.

Model simulations are provided by the numerical solutions to the relevant model ODEs, which are calculated using the ODE solver ode45 in MATLAB. We are interested in examining the dynamics of methylenomycin production in each of the 48 candidate models and therefore analyze the simulations of MMY provided by numerical solutions to the corresponding ODE (30).

### Available experimental data

Methylenomycin production by *S. coelicolor* has been shown to adopt a typical dynamical profile [19, 20]. Once expression is initiated, usually by environmental conditions that are thought to establish MMF production, it increases relatively quickly towards a global maximum level. Expression then decreases from this maximum, reaching a relatively low level at steady-state. This profile aligns with the premise that the system is initially held in a repressed state until MmfR is released by MMF to trigger methylenomycin expression, which then increases quickly until free MmfR and MmyR cause secondary repression and eventual equilibrium of the feedback loop.

We consider the binding affinity of MmfR to the *fpm* and *apm* to be strong, based on experimental data regarding binding interactions between a similar protein, SAV2270, and its associated DNA motifs (our unpublished data). We characterized the binding of this protein to Streptavidin Immobilized oligonucleotides using a Biocore T200 SPR instrument. Our data reveal that the association and dissociation rates of this protein:DNA binding are on the order of 10^5^ M ^−1^ *s* ^−1^ and 10^−2^ s ^−1^ respectively. As a result, we fix the model parameters relating to MmfR association and dissociation from both the *fpm* and *apm* at 10^5^ and 10^−2^ respectively (*k*_2_=*k*_5_=10^5^; *k*_−2_=*k*_−5_=10^−2^). The dimensionality of our experimental measurements agree with the corresponding parameters in our dimensional model and we are therefore able to apply these values directly. We assume that MmyR binding interactions are identical to that of MmfR and hence the same values are fixed for the parameters describing MmyR association and dissociation from the *fpm* and *apm* (*k*_1_=*k*_4_=10^5^; *k*_−1_=*k*_−4_=10^−2^).

Mutant strains of *S. coelicolor* that account for specific gene knockouts reveal qualitatively different methylenomycin production dynamics (Table 1). The mutant strain accounting for *mmyR* deletion, *Δ* *m* *m* *y* *R*, has been shown to exhibit increased methylenomycin expression compared to the wildtype; in the absence of MmyR, the overall capacity of the system to repress methylenomycin production is reduced and therefore the production of the antibiotic is increased. The *Δ* *m* *m* *f* *L* *H* *P* strain exhibits a complete cessation of methylenomycin expression; in the absence of the *mmfLHP* genes, the system is locked in the *apm*:MmfR complex since the expression of MmfR, MmyR and particularly MMF is prevented and thus the bound MmfR cannot be released. The *Δ* *mmfLHP*+ *Δ* *mmyR*+ *Δ* *mmfR* strain exhibits increased methylenomycin production compared to the wildtype; in the absence of MmfR and MmyR, both initially and as a result of any subsequent production by the *fpm*, the *apm* is able to produce methylenomycin in an unrestricted manner. The *Δ* *m* *m* *f* *L* *H* *P* strain with exogenous MMF exhibits relatively similar methylenomycin expression to that of the wildtype; in the absence of endogenous MMFs, exogenous MMF permits the release of MmfR and, in turn, methylenomycin expression. Experimentation with the *Δ* *m* *m* *f* *R* strain has thus far yielded inconclusive results and, as such, presents the opportunity for mathematical modeling simulations to inform future experimental studies.

**Table 1:** The effects of knocking out certain genes and combinations of genes observed experimentally, adapted from [9]

*S. coelicolor* strain	Methylenomycin production
Wildtype	+
*Δ* *mmyR*	+++
*Δ* *mmfLHP*	-
*Δ* *mmfLHP*+ *Δ* *mmyR*+ *Δ* *mmfR*	+++
*Δ* *mmfLHP*+exogenous MMF	+

The wildtype strain is allocated a single ‘+’ to denote typical methylenomycin expression. Over-expression and the cessation of expression are denoted by ‘+++’ and ‘-’ respectively

### Model selection via approximate Bayesian computation

**Fig. 3:**
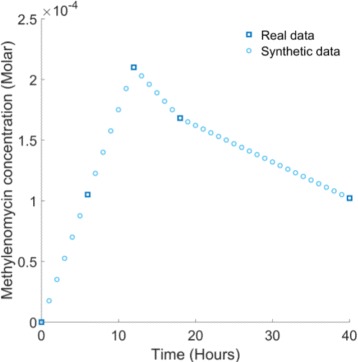
Experimental data representing current biological knowledge of typical methylenomycin expression in *S. coelicolor*. Real experimental data points taken from [20]. Synthetic data points are added uniformly between real data points to increase the rigor of model selection and parameter inference

In order to assess the potential of the 48 candidate architectures to reproduce the known characteristics of the system, we perform model selection based on approximate Bayesian computation (ABC) using the ABC-SysBio software package. ABC-SysBio combines Bayes’ rule with sequential Monte Carlo (SMC) approaches to solve parameter inference and model selection problems in systems biology [21–23]. The procedure determines the model, from a set of candidate models, that is most likely to have produced the associated experimental data. Extensive quantitative data regarding methylenomycin expression is lacking in the literature, however a time series expression profile is reported in [20]. We therefore provide ABC-SysBio with a dataset designed to replicate this profile (Fig. 3), with two important exceptions. Firstly, we specifically account for the dynamical series of data points in the 40 hour interval between hours 54 and 94 of the time series. This is because the 54 hour experimental time point is when methylenomycin expression commences and translates to the 0 hour time point in our simulations. The time points that precede 54 hours record the repression of methylenomycin production prior to the environmental trigger and are hence excluded when fitting a model that accounts purely for the dynamical response of the system. Secondly, we incorporate additional uniformly distributed ‘synthetic’ data points, increasing the size of the dataset from 5 points to 41, in order to provide a more rigorous data fitting task to the ABC-SysBio algorithm.

ABC-SysBio also requires a prior probability distribution on each model parameter subject to inference in order to establish the parameter space within which to locate acceptable parameter sets. The prior distributions chosen for all parameters associated with each of the 48 candidate models are uniform distributions on the interval [10^−4^,10^4^], that is, all candidate models are given an equal parameter space in attempting to identify parameter values capable of replicating our experimental dataset. We consider uniform priors to be the most suitable for model selection given the complete uncertainty surrounding the parameters subject to inference, and hence all potential parameter values require an equal probability of selection. We also impose prior distributions on the initial conditions of the necessary state variables due to the lack of experimental data regarding the physical quantity of DNA in the system: the prior distributions are uniform distributions on the interval [0,1] and are assigned only to the MmfR:*fpm* and MmfR:*apm* complexes, all other initial conditions are set equal to zero. ABC-SysBio convergence is dependent on the sequential satisfaction of a predefined series of decreasing error thresholds by a predefined number of solutions (see Methods). Here, the number of solutions required to satisfy each error threshold is 500 [24] in order to reduce the time frame required for convergence; the number of models subject to selection coupled with the inability to parallelize the process presents a particularly time consuming computational workload. The user-defined error function designed to measure the accuracy of simulations takes the mean absolute value of the difference between model outputs and data values:

32 $$\begin{array}{*{20}l} E = \frac{1}{41}\sum\limits_{i=1}^{41} |x_{i}-d_{i}|, \end{array} $$

where *E* is the error and *x*_*i*_, *d*_*i*_ are the model outputs and data values at each of the 41 corresponding time points, *t*_*i*_, respectively.

**Fig. 4:**
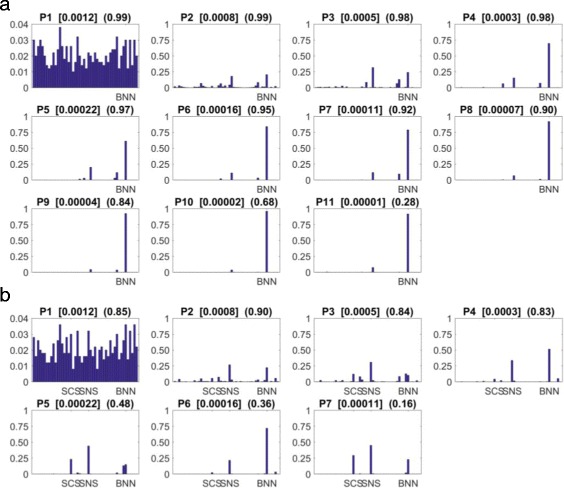
ABC-SysBio model selection results. **a** Histograms showing the probabilities of producing the full dataset for the 48 candidate models. **b** Histograms showing the probabilities of producing the real experimental dataset for the 48 candidate models. The numbers above each histogram denote the population number, the error threshold *ε* (*square brackets*) and the acceptance rate (*parentheses*) respectively. The number of accepted solutions required to satisfy each error threshold is 500

The results of our model selection are shown in Fig. 4. The final probability distributions reveal that the model most likely to have produced the experimental data is BNN, the model formulated based on our current knowledge (Fig. 4a). The BNN model achieved a 0.916 probability of producing our data which is vastly superior to the remaining models, 36 of which were statistically eliminated through the selection process. This suggests that the most plausible network of molecular interactions underlying this system should account for MMF-MmfR interactions both at existing DNA:MmfR complexes and in solution, no MMY-MmfR interactions at all and no MMY-MmyR interactions at all, as depicted in Fig. 2.

In order to verify that the addition of synthetic data points does not restrict the emergence of other viable candidate models, we repeated the model selection procedure using only the 5 real experimental data points taken from [20]. Mean absolute error generally increases with decreasing numbers of data points which subsequently increases the difficulty for each population of solutions to meet the same error thresholds. Hence, the acceptance rate decreases and the process becomes more time consuming; this run took longer than the original run and met 7 thresholds compared to the previous 11 (Fig. 4b). The probability distribution across all models clearly identified the most likely models as early as P2, which converged further at P4 and P6 to suggest that BNN was a likely model architecture, in agreement with our initial result. However, P3, P5 and P7 identified a different distribution which suggested that models SCS and SNS were also likely candidates. Given that ABC-SysBio appeared to present two alternating probability distributions, it is probable that additional local minima were identified in this case. To further investigate the set of plausible models identified using this Bayesian inference framework, we next employed global optimization methods, as well as analysis of mutant versions of the candidate models, as described in the following sections.

### Parameter inference via global optimization

**Fig. 5:**
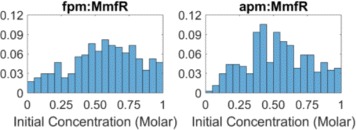
ABC-SysBio parameter inference results. Histograms show the probability distributions on the two parameters describing the initial concentration of the fpm:MmfR and apm:MmfR complexes

ABC-SysBio performs parameter inference by producing probability distributions on the numerical values that comprise accepted parameter sets during the model selection process. For example, the distributions on the initial conditions imposed on the *fpm*:MmfR and *apm*:MmfR complexes reveal that statistically these values can be approximated to be 0.6 and 0.5 respectively (Fig. 5). These distributions are insightful, but cannot provide complete clarity over the numerical values inferred in all cases. Other parameter inference methods, such as global optimization, place greater focus on the identification of specific numerical parameter sets capable of minimizing the user-defined error function. The genetic algorithm (GA), is a particularly powerful global optimization tool and is exploited regularly in biological model parameter inference [25, 26]. The GA converges to the solution providing the global minimum error within the allocated parameter space by evolving an initial population of randomly generated solutions over a large number of generations. This process is based on natural selection, giving the best solutions in the population the best chance of creating the next generation of solutions (see Methods).

**Fig. 6:**
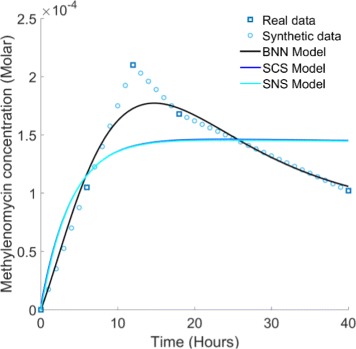
Genetic algorithm global optimization results. The BNN model is able to fit the experimental data using the optimal parameter set identified by the GA. The absolute mean error provided by this optimal solution is 6.12 ×10^−6^. The optimal fits provided by the SCS and SNS models are similar and are not as accurate as the BNN model. The absolute mean error provided by both of these optimal solutions is 2.39 ×10^−5^

In our case, the error function minimized by the GA is the same absolute mean error function used for ABC-SysBio model selection (32). We also allocate the same parameter space to the GA by imposing lower and upper bounds on the inferred parameters of 10^−4^ and 10^4^ respectively. Again, the initial conditions imposed on the model variables are zero with the exception of those regarding the *fpm*:MmfR and *apm*:MmfR complexes which we approximate to be 0.55 in light of our ABC-SysBio probability distributions and given that we require both initial concentrations to be equal. The results of our global optimization are shown in Fig. 6. The BNN model is capable of accurately matching the experimental time-course data when optimized within the same parameter space used for model selection. The optimal parameter set identified by the GA is listed in Table 2 and provides an absolute mean error of 6.12×10^−6^. The four parameter values describing protein degradation (*γ*_1,2,3,4_) vary by one order of magnitude at most; the remaining parameter values all describe protein:protein association and dissociation and vary by three orders of magnitude at most. Hence, we conclude that the numerical ranges of these optimal parameter values are reasonable within this biological context.

**Table 2:** Optimal parameter values for our BNN model

Reaction	Value (M ^−1^ *s* ^−1^)	Reaction	Value (s ^−1^)
*k*_3_	3.6119	*k*_−3_	0.1092
*k*_6_	0.9079	*k*_−6_	5.7766
*k*_14_	0.0065	*k*_−14_	0.2208
−	−	*k*_7_	2.6978
−	−	*k*_8_	0.8902
−	−	*k*_9_	5.8903
−	−	*k*_10_	0.1101
−	−	*k*_11_	0.6296
−	−	*k*_12_	0.5307
−	−	*k*_13_	0.0880
−	−	*γ*_1_	0.9470
−	−	*γ*_2_	2.7057
−	−	*γ*_3_	0.2248
−	−	*γ*_4_	0.1646

This optimal parameter set is dimensional, with parameters in the first and second reaction columns taking the units M ^−1^ *s* ^−1^ and s ^−1^ respectively based on standard mass action kinetics

To investigate further, we also optimized the parameters of the SCS and SNS models against the experimental data using the GA, in an identical manner to that previously done for the BNN model. This revealed that neither model was able to achieve the same quality of fit to the data as the BNN (minimum error of 2.39×10^−5^ for both SCS and SNS compared to 6.12×10^−6^ for BNN). In addition, neither the SCS or SNS models were able to even qualitatively replicate the non-monotonicity in the response that is clearly exhibited in the experimental data.

### Monte Carlo simulations of methylenomycin production in mutant strains

We performed additional model validation by testing the BNN model against our qualitative data regarding methylenomycin production in mutant *S. coelicolor* strains. We employ Monte Carlo simulations to examine methylenomycin production under four distinct conditions corresponding to the mutant strains described in Table 1. By examining the dynamical response to specific gene knockouts against the wildtype strain, represented by the optimal BNN model output in Fig. 6, we are able to investigate the qualitative effect of adapting our BNN model to emulate these mutant strains.

When simulating MMY production in the different mutant strains, we account for *Δ* *mmyR* by simply setting the parameter describing MmyR production from the *fpm*, *k*_7_, to zero. However, *Δ* *mmfR* strains are incapable of producing MmfR and therefore cannot be simulated in the initial repressed state comprised of the *fpm*:MmfR and *apm*:MmfR complexes. Hence, the parameter describing MmyR production from the *fpm*, *k*_8_, is set to zero and the allocation of initial concentrations is adapted to exclude the *fpm*:MmfR and *apm*:MmfR complexes. The *Δ* *mmfLHP* strain is simulated by setting the initial concentration of the *fpm* and its associated complexes to zero, since the entire DNA module has been knocked out. The addition of exogenous MMF involves allocating this variable an initial concentration of 0.55 to align with the initial concentrations allocated to the relevant variables, that is, no new model parameters are introduced to describe production of exogenous MMF. Mutant strains comprising combinations of gene knockouts are simulated by combining the appropriate adaptations.

Specifically, in order to simulate the *Δ* *mmyR* strain we set *k*_7_=0. To simulate the *Δ* *mmfLHP* strain the initial concentration of 0.55 is imposed on the *apm*:MmfR complex only, all other initial concentrations are set equal to zero. To simulate the *Δ* *mmfLHP*+ *Δ* *mmyR*+ *Δ* *mmfR* strain we set *k*_7_=*k*_8_=0 and all initial concentration are set equal to zero with the exception of the *apm* which is set equal to 0.55. To simulate the *Δ* *mmfLHP*+exogenous MMF strain initial concentrations of the *apm* and MMF are set equal to 0.55, and all other initial concentrations are set equal to zero.

**Fig. 7:**
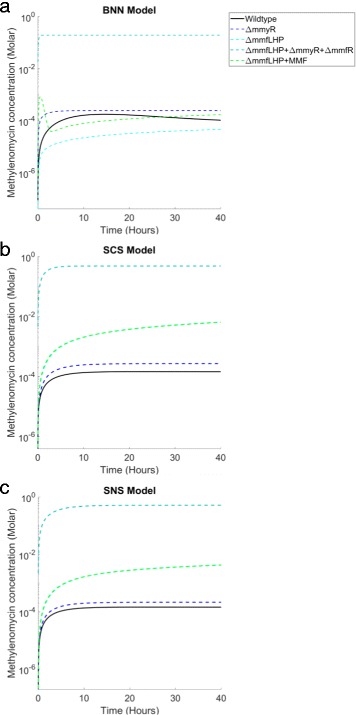
Monte Carlo simulation results. **a** The BNN model is able to simulate the qualitative data regarding four mutant S. coelicolor strains when adapted to replicate the corresponding gene knockouts. **b** The SCS model is unable to simulate qualitative data regarding the *Δ*mmfLHP and *Δ*mmfLHP+MMF knockout strains. **c** The SNS model is unable to simulate qualitative data regarding the *Δ*mmfLHP and *Δ*mmfLHP+MMF knockout strains

Monte Carlo simulations assign random values in the interval [10^−4^,10^4^] to all model parameters, excluding those that retain their fixed values assigned for previous model selection and parameter inference purposes, as we continue to examine dimensional dynamic responses. We run a total of 10^4^ Monte Carlo simulations to allow for substantial sampling of the parameter space within a feasible time frame. Each simulation outputs MMY production for each of the four mutant strains and calculates the ratio of the mean value to that of the optimal wildtype simulation. We utilize these ratios to investigate the ability of our model to satisfy the following four criteria, which capture the experimentally observed responses of the mutant strains:

1. $\frac {\Delta mmyR}{\text {wildtype}} > 1.1$,
2. $\frac {\Delta mmfLHP}{\text {wildtype}} < 0.9$,
3. $\frac {\Delta mmfLHP+\Delta mmyR+\Delta mmfR}{\text {wildtype}} > 1.1$,
4. $0.9 < \frac {\Delta mmfLHP+\text {MMF}}{\text {wildtype}} < 1.1$,

where overproduction translates to an increase in mean MMY production of <10*%*, cessation translates to a decrease in MMY production of <10*%* and comparable production translates to a maximum increase or decrease in MMY production of less than 10%. The results of our Monte Carlo simulations are shown in Fig. 7. Parameter sets were identified for BNN that are capable of satisfying each of the four criteria, within the same dimensional solution space as the optimized wildtype model (Fig. 7a). Given the uncertainty regarding the effect of gene knockouts on the reaction kinetics and MMY production of the system, this qualitative agreement offers further validation of the replication and prediction capabilities of the BNN model.

The SCS and SNS models are also able to simulate the responses observed experimentally for the *Δ* *mmyR* and the *Δ* *mmfLHP*+ *Δ* *mmyR*+ *Δ* *mmfR* knockout strains, but not for the *Δ* *mmfLHP* and *Δ* *mmfLHP*+exogenous MMF knockouts (Fig. 7b and 7c). This is likely due to the most significant mechanistic property separating them from BNN, i.e. the interaction of MMY with one or both of MmyR and MmfR. This interaction results in decreased repression of the *apm* since the MMY is negating the action of either or both regulators and hence the *apm* is less restricted in producing MMY, which causes an overproduction of the antibiotic for the *Δ* *mmfLHP* and *Δ* *mmfLHP*+exogenous MMF knockouts that has not been observed experimentally (Fig. 7b and 7c). We therefore conclude that the BNN model remains the most likely candidate model to explain all the available experimental data for this system.

### Experimental design for future studies

We are able to inform the design of future experimental studies in light of our results. For example, we are interested in quantifying the response of the *Δ* *m* *m* *y* *R* and *Δ* *m* *m* *f* *L* *H* *P*+*Δ* *m* *m* *y* *R*+*Δ* *m* *m* *f* *R* strains in order to verify our model prediction that the five gene mutant elicits a more rapid and significantly greater overproduction of MMY. This has implications both in terms of improving product yields for industrially relevant natural products, and also regarding the potential adverse effects this might cause in the cells, such as toxicity. The result of these experiments would subsequently reveal whether the *Δ* *m* *m* *f* *L* *H* *P*+*Δ* *m* *m* *y* *R*+*Δ* *m* *m* *f* *R* is the most effective knockout for improving antibiotic production in novel synthetic regulatory systems.

In the event that directly quantifying MMY production is inconclusive, we would be interested in replacing the gene controlled by the *apm* with a reporter gene coding for fluorescence or luminescence such as green fluorescent protein (GFP) or *lux* genes respectively. This output may enable us to measure the response of the different mutant strains with greater clarity, since experiments of this nature are already well characterized, particularly in the related bacterium *S. venezuelae*.

Finally, we are also interested in examining the *Δ* *m* *m* *f* *L* *H* *P*+MMF mutant in order to establish the quantity of exogenous MMF and the specific time point of induction that provides optimal MMY production. Our model predicts a narrow production window for this strain which may suggest that direct MMY quantification is not straightforward and that, again, experimental designs incorporating reporter genes would provide improved results.

## Conclusions

We have developed a plausible model architecture for the regulatory system controlling methylenomycin production in *Streptomyces coelicolor*. This architecture was found to most closely reproduce the various dynamical responses described by experimental time series data for this system, when tested against 47 other candidate architectures. Global optimization of the model parameters produced close agreement with the experimental data. Appropriate adjustments to the proposed model architecture allow it to replicate observed changes in the dynamics of methylenomycin production in a number of mutant *S. coelicolor* strains.

The mechanistic details captured in the proposed regulatory architecture provide useful insights for the design of future experiments to further investigate the operation of this system, and demonstrate the potential of mathematical models to elucidate the design principles of complex biological control systems. We expect that the emergence of further quantitative experimental data for this system will inform further model development and validation, and allow for the generation of optimized models that are capable of accurately predicting the dynamical responses of one of the most prevalent and important gene regulatory networks in nature.

## Methods

### ABC-SysBio model selection

ABC-SysBio is a Python software package that is designed specifically for parameter inference and model selection in biological systems research using the approach of approximate Bayesian computation (ABC) [21]. The program enables ABC inference of mathematical models via sequential Monte Carlo (SMC) approaches [21–23]. Monte Carlo approaches to computational simulations involve generating random candidate solutions, testing their fitness against a desired output and repeating until a viable solution can be identified. In this way, vast numbers of randomly selected parameter sets can be examined in building an accurate approximation to the posterior distribution defined by conditional probabilities known as Bayes’ theorem:

33 $$\begin{array}{*{20}l} P(A|B) = \frac{P(B|A)P(A)}{P(B)}, \end{array} $$

where *P*(*B*)>0. The ABC-SMC approach proceeds in the following manner: the first ‘population’ of accepted solutions or ‘particles’ is generated randomly based on the prior distributions imposed on the model parameters. Each particle gives rise to a simulated dataset, *D* ^⋆^, which is compared to the fixed experimental dataset, *D*, by an appropriate distance function and its fitness is scored accordingly. Acceptance of a particle is dependent on a decreasing sequence of error thresholds, *ε*, set to correspond with each population. That is,

34 $$\begin{array}{*{20}l} d(D^{\star},D) < \epsilon_{i}, \end{array} $$

where *ε*_1_>*ε*_2_>… >*ε*_*n*_ and *d* is the distance function. Each subsequent population is obtained by perturbing particles from the previous population in accordance with a predetermined perturbation kernel, proceeding until the model is unable to produce particles of sufficient fitness to satisfy the immediate threshold.

An array of model-specific criteria are required to allow the ABC-SysBio package to run efficiently: the sequence of decreasing error thresholds, *ε*, must be provided whereby only the particles capable of providing error less than that of the threshold will be accepted by the algorithm. Each *ε* must be satisfied in succession until the particles are unable to satisfy the next threshold. Satisfaction of an individual threshold is dependent on the number of particles accepted; the number of acceptable particles required before passage to the next threshold must also be predetermined. The larger the number of particles, the higher probability of significant inference and the longer the duration of algorithm to reach convergence. Each individual parameter subject to inference requires a prior probability distribution in order to establish the parameter space within which to locate acceptable particles. Sequences of numerical values representing the relevant experimental data and the corresponding time points must also be provided; the number of data points and time points must be equal. Time series data is currently the only supported data format. One or more distinct datasets can be supplied and can be fitted to any individual model variable or combination of variables. Convergence of the algorithm is dependent on all of the aforementioned factors and hence it may require several trials to establish the appropriate performance criteria. To achieve credible results, it is advised that identical parameter inference and model selection tasks are repeated multiple times due to the random nature of the Monte Carlo simulations that drive the algorithm. Note that all models submitted to the ABC-SysBio package must be written in Systems Biology Markup Language (SBML), a systems biology programming language based on Extensible Markup Language (XML).

### Global optimization

We employ the genetic algorithm function in MATLAB in order to optimize our model against our experimental data. The reaction rate constants *k*_*i*_ are chosen as optimization variables with the exception of those fixed in light of our kinetic data. The GA mimics natural selection; converging to the global minimum within the allocated parameter space by evolving an initial population of randomly generated solutions over a large number of generations. The probability of obtaining the global optimum solution is maximized by selecting the largest population size and number of generations possible. However, increasing the computational workload in this manner also greatly increases the time frame required for the algorithm to converge. Establishing an effective compromise is key for successful deployment. We ran the GA under the following conditions:

- Population: 1000
- Generations: 1000
- Bounds imposed on parameter values: [ 10^−4^, 10^4^]

The default GA population size in MATLAB is 200 for inference of over 5 parameters however, we selected a population size of 1000 for inference of 17 parameters since a single time series dataset presents a relatively low computational workload and thus allows optimal solutions for large populations to be obtained in feasible time frames. We selected a large parameter space due to our focus on establishing optimal model performance in light of the lack of documentation regarding reaction rates.

## Abbreviations

ABC: approximate Bayesian computation
apm: antibiotic producing module
DNA: deoxyribonucleic acid
fpm: furan producing module
GA: genetic algorithm
GFP: green fluorescent protein
MARE: methylenomycin auto-regulatory response element
MMF: methylenomycin furan
MMY: methylenomycin
ODE: ordinary differential equation
SBML: Systems biology markup language
SMC: Sequential Monte Carlo
S. coelicolor: Streptomyces coelicolor
XML: Extensible markup language
